# Crystal structure of 5-chloro-2-(3-fluoro­phen­yl)-3-methyl­sulfinyl-1-benzo­furan

**DOI:** 10.1107/S1600536814017966

**Published:** 2014-08-09

**Authors:** Hong Dae Choi, Uk Lee

**Affiliations:** a Department of Chemistry, Dongeui University, San 24 Kaya-dong, Busanjin-gu, Busan 614-714, Republic of Korea; b Department of Chemistry, Pukyong National University, 599-1 Daeyeon 3-dong, Nam-gu, Busan 608-737, Republic of Korea

**Keywords:** crystal structure, benzo­furan, 3-fluoro­phen­yl, C—H⋯O hydrogen bonds

## Abstract

In the title compound, C_15_H_10_ClFO_2_S, the dihedral angle between the plane of the benzo­furan ring system [r.m.s. deviation = 0.013 (1) Å] and that of the 3-fluoro­phenyl ring [r.m.s. deviation = 0.005 (1) Å] is 31.36 (5)°. In the crystal, mol­ecules are linked by two different pairs of C—H⋯O hydrogen bonds, forming inversion dimers.

## Related literature   

For the pharmaceutical properties of compounds containing the benzo­furan moiety, see: Aslam *et al.* (2009 ▶); Choi *et al.* (2003 ▶); Galal *et al.* (2009 ▶); Khan *et al.* (2005 ▶); Ono *et al.* (2002 ▶). For natural products with a benzo­furan ring, see: Akgul & Anil (2003 ▶); Soekamto *et al.* (2003 ▶). For the synthesis of the starting material 5-chloro-2-(3-fluoro­phen­yl)-3-methyl­sulf­an­yl-1-benzo­furan, see: Choi *et al.* (1999 ▶). For a related structure, see: Choi *et al.* (2009 ▶).
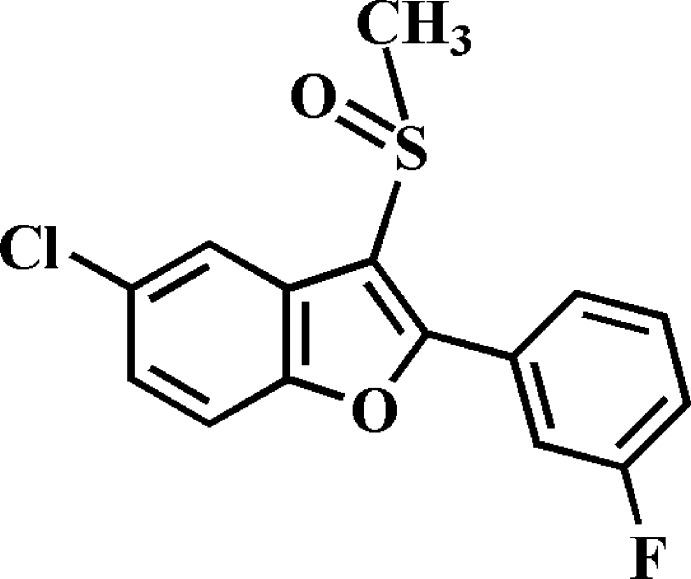


## Experimental   

### Crystal data  

C_15_H_10_ClFO_2_S
*M* *_r_* = 308.74
Triclinic, 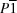

*a* = 8.0038 (1) Å
*b* = 8.4322 (1) Å
*c* = 10.6782 (2) Å
α = 88.933 (1)°
β = 81.008 (1)°
γ = 66.859 (1)°
*V* = 653.81 (2) Å^3^
*Z* = 2
Mo *K*α radiation
μ = 0.46 mm^−1^
*T* = 173 K
0.47 × 0.34 × 0.33 mm

### Data collection  

Bruker SMART APEXII CCD diffractometer
Absorption correction: multi-scan (*SADABS*; Bruker, 2009 ▶) *T*_min_ = 0.813, *T*_max_ = 0.863
11737 measured reflections
3124 independent reflections
2887 reflections with *I* > 2σ(*I*)
*R*_int_ = 0.022

### Refinement  

*R*[*F* ^2^ > 2σ(*F* ^2^)] = 0.032
*wR*(*F* ^2^) = 0.086
*S* = 1.07
3124 reflections
182 parameters
H-atom parameters constrained
Δρ_max_ = 0.30 e Å^−3^
Δρ_min_ = −0.44 e Å^−3^

### 

Data collection: *APEX2* (Bruker, 2009 ▶); cell refinement: *SAINT* (Bruker, 2009 ▶); data reduction: *SAINT*; program(s) used to solve structure: *SHELXS97* (Sheldrick, 2008 ▶); program(s) used to refine structure: *SHELXL97* (Sheldrick, 2008 ▶); molecular graphics: *ORTEP-3 for Windows* (Farrugia, 2012 ▶) and *DIAMOND* (Brandenburg, 1998 ▶); software used to prepare material for publication: *SHELXL97*.

## Supplementary Material

Crystal structure: contains datablock(s) I. DOI: 10.1107/S1600536814017966/zq2226sup1.cif

Structure factors: contains datablock(s) I. DOI: 10.1107/S1600536814017966/zq2226Isup2.hkl

Supporting information file. DOI: 10.1107/S1600536814017966/zq2226Isup3.cml

**Fig. 1.**
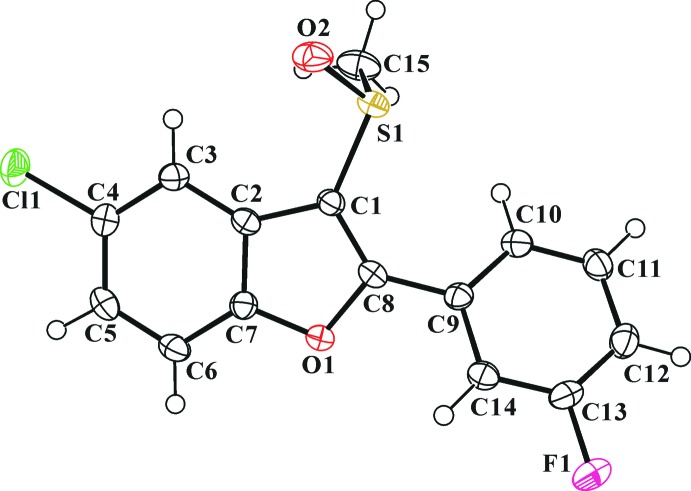
The molecular structure of the title molecule with the atom numbering scheme The displacement ellipsoids are drawn at the 50% probability level. The hydrogen atoms are presented as small spheres of arbitrary radius.

**Fig. 2.**
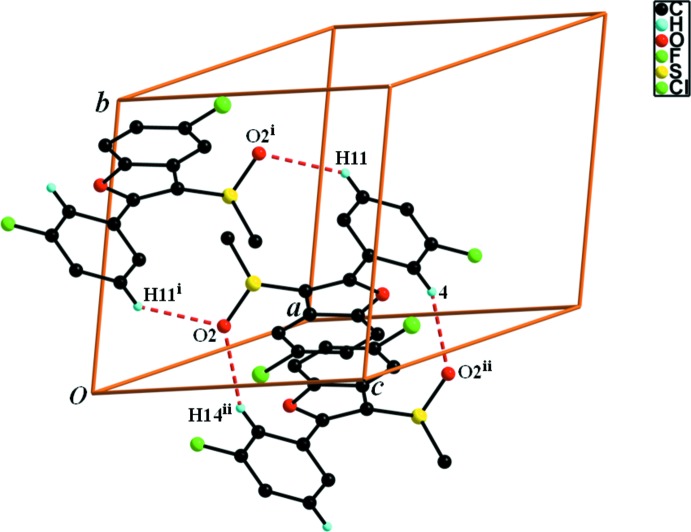
A view of the C—H···O hydrogen bonds (dotted lines) in the crystal structure of the title compound. H atoms non-participating in hydrogen-bonding were omitted for clarity [symmetry codes: (i) - x, - y + 1, - z + 1; (ii) - x + 1, - y, - z + 1].

. DOI: 10.1107/S1600536814017966/zq2226fig1.tif
The mol­ecular structure of the title mol­ecule with the atom numbering scheme The displacement ellipsoids are drawn at the 50% probability level. The hydrogen atoms are presented as small spheres of arbitrary radius.

x y z x y z . DOI: 10.1107/S1600536814017966/zq2226fig2.tif
A view of the C—H⋯O hydrogen bonds (dotted lines) in the crystal structure of the title compound. H atoms non-participating in hydrogen-bonding were omitted for clarity [symmetry codes: (i) − *x*, − *y* + 1, − *z* + 1; (ii) − *x* + 1, − *y*, − *z* + 1].

CCDC reference: 1017893

Additional supporting information:  crystallographic information; 3D view; checkCIF report

## Figures and Tables

**Table 1:** Hydrogen-bond geometry (Å, °)

*D*—H⋯*A*	*D*—H	H⋯*A*	*D*⋯*A*	*D*—H⋯*A*
C11—H11⋯O2^i^	0.95	2.57	3.3470 (18)	139
C14—H14⋯O2^ii^	0.95	2.59	3.4884 (17)	157

Symmetry codes: (i) 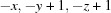; (ii) 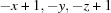.

## supplementary crystallographic information

### S1. Comment

Recently, a number of benzofuran compounds have drawn much attention owing to their interesting pharmaceutical properties such as antibacterial and antifungal, antitumor and antiviral, antimicrobial activities (Aslam *et al.* 2009; Galal *et al.*, 2009; Khan *et al.*, 2005), and potential inhibitors of β-amyloid formation (Choi *et al.*, 2003, Ono *et al.*, 2002). These benzofuran derivatives occur in a wide range of natural products (Akgul & Anil, 2003; Soekamto *et al.*, 2003). As a part of our ongoing project of 2-aryl-5-chloro-3-methylsulfinyl-1-benzofuran derivatives containing 4-fluorophenyl substituent in 2-position (Choi *et al.*, 2009), we report herein on the crystal structure of the title compound.

In the title molecule (Fig. 1), the benzofuran unit is essentially planar, with a mean deviation of 0.013 (1) Å from the least-squares plane defined by the nine constituent atoms. The 3-fluorophenyl ring is essentially planar, with a mean deviation of 0.005 (1) Å from the least-squares plane defined by the six constituent atoms. The dihedral angle formed by the benzofuran ring system and the 3-fluorophenyl ring is 31.36 (5)°. In the crystal structure (Fig. 2), molecules are linked by two different pairs of C—H···O hydrogen bonds (Table 1), forming inversion dimers.

### S2. Experimental

The starting material 5-chloro-2-(3-fluorophenyl)-3-methylsulfanyl-1-benzofuran was prepared by literature method (Choi *et al.* 1999). 3-Chloroperoxybenzoic acid (77%, 269 mg, 1.2 mmol) was added in small portions to a stirred solution of the starting material (322 mg, 1.1 mmol) in dichloromethane (30 mL) at 273 K. After being stirred at room temperature for 6h, the mixture was washed with saturated sodium bicarbonate solution (2 X 20 mL) and the organic layer was separated, dried over magnesium sulfate, filtered and concentrated at reduced pressure. The residue was purified by column chromatography (hexane–ethyl acetate, 1:1 *v*/*v*) to afford the title compound as a colorless solid [yield 73% (248 mg); m.p. 483–484 K; *R*_f_ = 0.48 (hexane-ethyl acetate, 1:1 *v*/*v*)]. Single crystals suitable for X-ray diffraction were prepared by slow evaporation of a solution of the title compound (120 mg) in acetone (20 mL) at room temperature.

### S3. Refinement

All H atoms were positioned geometrically and refined using a riding model, with C—H = 0.95 Å for aryl and 0.98 Å for methyl H atoms, *U*_iso_ (H) = 1.2*U*_eq_ (C) for aryl and 1.5*U*_eq_ (C) for methyl H atoms.The positions of methyl hydrogens were optimized using the SHELXL-97's command AFIX 137 (Sheldrick, 2008).

### Crystal data

**Table:**

C_15_H_10_ClFO_2_S	*Z* = 2
*M**_r_* = 308.74	*F*(000) = 316
Triclinic, *P*1	*D*_x_ = 1.568 Mg m^−^^3^
Hall symbol: -P 1	Mo *K*α radiation, λ = 0.71073 Å
*a* = 8.0038 (1) Å	Cell parameters from 6836 reflections
*b* = 8.4322 (1) Å	θ = 2.6–28.5°
*c* = 10.6782 (2) Å	µ = 0.46 mm^−^^1^
α = 88.933 (1)°	*T* = 173 K
β = 81.008 (1)°	Block, colourless
γ = 66.859 (1)°	0.47 × 0.34 × 0.33 mm
*V* = 653.81 (2) Å^3^	

### Data collection

**Table:**

Bruker SMART APEXII CCD diffractometer	3124 independent reflections
Radiation source: rotating anode	2887 reflections with *I* > 2σ(*I*)
Graphite multilayer monochromator	*R*_int_ = 0.022
Detector resolution: 10.0 pixels mm^-1^	θ_max_ = 28.0°, θ_min_ = 1.9°
φ and ω scans	*h* = −10→10
Absorption correction: multi-scan (*SADABS*; Bruker, 2009)	*k* = −11→11
*T*_min_ = 0.813, *T*_max_ = 0.863	*l* = −14→13
11737 measured reflections	

### Refinement

**Table:**

Refinement on *F*^2^	Primary atom site location: structure-invariant direct methods
Least-squares matrix: full	Secondary atom site location: difference Fourier map
*R*[*F*^2^ > 2σ(*F*^2^)] = 0.032	Hydrogen site location: difference Fourier map
*wR*(*F*^2^) = 0.086	H-atom parameters constrained
*S* = 1.07	*w* = 1/[σ^2^(*F*_o_^2^) + (0.0458*P*)^2^ + 0.2424*P*] where *P* = (*F*_o_^2^ + 2*F*_c_^2^)/3
3124 reflections	(Δ/σ)_max_ < 0.001
182 parameters	Δρ_max_ = 0.30 e Å^−^^3^
0 restraints	Δρ_min_ = −0.44 e Å^−^^3^

### Special details

**Table:**

Experimental. 1H NMR (δ p.p.m., CDCl3, 400 Hz): 8.23 (d, J = 2.04 Hz, 1H), 7.62 (d, J = 7.88 Hz, 1H), 7.54-7.58 (m, 1H), 7.45-7.52 (m, 2H), 7.38 (dd, J = 8.88 and 2.04 Hz, 1H), 7.16-7.22 (m, 1H), 3.11 (s, 3H).
Geometry. All esds (except the esd in the dihedral angle between two l.s. planes) are estimated using the full covariance matrix. The cell esds are taken into account individually in the estimation of esds in distances, angles and torsion angles; correlations between esds in cell parameters are only used when they are defined by crystal symmetry. An approximate (isotropic) treatment of cell esds is used for estimating esds involving l.s. planes.
Refinement. Refinement of F^2^ against ALL reflections. The weighted R-factor wR and goodness of fit S are based on F^2^, conventional R-factors R are based on F, with F set to zero for negative F^2^. The threshold expression of F^2^ > 2sigma(F^2^) is used only for calculating R-factors(gt) etc. and is not relevant to the choice of reflections for refinement. R-factors based on F^2^ are statistically about twice as large as those based on F, and R- factors based on ALL data will be even larger.

### Fractional atomic coordinates and isotropic or equivalent isotropic displacement parameters (Å^2^)

**Table:**

	*x*	*y*	*z*	*U*_iso_*/*U*_eq_	
Cl1	0.82187 (5)	−0.13963 (5)	−0.02027 (3)	0.03515 (11)	
S1	0.21038 (4)	0.30346 (4)	0.39543 (3)	0.02300 (10)	
F1	0.53226 (14)	0.29209 (13)	0.95736 (8)	0.0395 (2)	
O1	0.70635 (13)	0.13881 (12)	0.49383 (9)	0.0234 (2)	
O2	0.17787 (15)	0.16956 (13)	0.32625 (11)	0.0325 (2)	
C1	0.44633 (17)	0.21871 (16)	0.40865 (12)	0.0210 (3)	
C2	0.59652 (17)	0.10631 (16)	0.31628 (12)	0.0210 (3)	
C3	0.61509 (18)	0.04393 (17)	0.19238 (13)	0.0231 (3)	
H3	0.5120	0.0719	0.1499	0.028*	
C4	0.79058 (19)	−0.06027 (18)	0.13484 (13)	0.0252 (3)	
C5	0.94567 (19)	−0.10505 (18)	0.19490 (14)	0.0275 (3)	
H5	1.0635	−0.1775	0.1510	0.033*	
C6	0.92781 (19)	−0.04413 (18)	0.31773 (14)	0.0265 (3)	
H6	1.0308	−0.0728	0.3605	0.032*	
C7	0.75209 (18)	0.06058 (16)	0.37470 (13)	0.0221 (3)	
C8	0.51944 (17)	0.23358 (16)	0.51261 (13)	0.0214 (3)	
C9	0.44315 (18)	0.32961 (17)	0.63441 (12)	0.0216 (3)	
C10	0.29269 (19)	0.48798 (18)	0.64645 (13)	0.0256 (3)	
H10	0.2383	0.5349	0.5740	0.031*	
C11	0.2218 (2)	0.57761 (18)	0.76298 (14)	0.0281 (3)	
H11	0.1182	0.6846	0.7703	0.034*	
C12	0.3016 (2)	0.51149 (19)	0.86908 (14)	0.0296 (3)	
H12	0.2542	0.5717	0.9495	0.036*	
C13	0.4517 (2)	0.35607 (19)	0.85427 (13)	0.0270 (3)	
C14	0.52576 (18)	0.26207 (17)	0.74107 (13)	0.0235 (3)	
H14	0.6293	0.1551	0.7350	0.028*	
C15	0.2148 (2)	0.4624 (2)	0.28245 (17)	0.0365 (4)	
H15A	0.3053	0.4062	0.2070	0.055*	
H15B	0.2488	0.5481	0.3205	0.055*	
H15C	0.0927	0.5199	0.2581	0.055*	

### Atomic displacement parameters (Å^2^)

**Table:**

	*U*^11^	*U*^22^	*U*^33^	*U*^12^	*U*^13^	*U*^23^
Cl1	0.0340 (2)	0.0413 (2)	0.02564 (19)	−0.01237 (16)	0.00258 (14)	−0.00911 (14)
S1	0.01657 (16)	0.02380 (17)	0.02762 (18)	−0.00635 (12)	−0.00525 (12)	0.00218 (12)
F1	0.0448 (6)	0.0467 (5)	0.0251 (4)	−0.0123 (4)	−0.0156 (4)	0.0027 (4)
O1	0.0190 (4)	0.0254 (5)	0.0236 (5)	−0.0056 (4)	−0.0057 (4)	−0.0006 (4)
O2	0.0296 (5)	0.0282 (5)	0.0451 (6)	−0.0133 (4)	−0.0166 (5)	0.0030 (4)
C1	0.0169 (6)	0.0214 (6)	0.0233 (6)	−0.0058 (5)	−0.0038 (5)	0.0009 (5)
C2	0.0181 (6)	0.0204 (6)	0.0238 (6)	−0.0069 (5)	−0.0034 (5)	0.0025 (5)
C3	0.0220 (6)	0.0244 (6)	0.0232 (6)	−0.0091 (5)	−0.0048 (5)	0.0016 (5)
C4	0.0268 (7)	0.0245 (6)	0.0236 (6)	−0.0104 (5)	−0.0008 (5)	−0.0017 (5)
C5	0.0206 (6)	0.0255 (6)	0.0316 (7)	−0.0058 (5)	0.0007 (5)	−0.0016 (5)
C6	0.0189 (6)	0.0257 (6)	0.0326 (7)	−0.0058 (5)	−0.0057 (5)	0.0008 (5)
C7	0.0213 (6)	0.0213 (6)	0.0233 (6)	−0.0077 (5)	−0.0045 (5)	0.0009 (5)
C8	0.0178 (6)	0.0201 (6)	0.0248 (6)	−0.0058 (5)	−0.0039 (5)	0.0021 (5)
C9	0.0205 (6)	0.0231 (6)	0.0226 (6)	−0.0099 (5)	−0.0044 (5)	0.0005 (5)
C10	0.0248 (7)	0.0250 (6)	0.0265 (7)	−0.0082 (5)	−0.0067 (5)	0.0018 (5)
C11	0.0252 (7)	0.0249 (6)	0.0306 (7)	−0.0065 (5)	−0.0026 (5)	−0.0036 (5)
C12	0.0303 (7)	0.0326 (7)	0.0259 (7)	−0.0132 (6)	−0.0019 (6)	−0.0052 (5)
C13	0.0296 (7)	0.0330 (7)	0.0231 (7)	−0.0155 (6)	−0.0095 (5)	0.0040 (5)
C14	0.0214 (6)	0.0246 (6)	0.0258 (7)	−0.0096 (5)	−0.0055 (5)	0.0017 (5)
C15	0.0326 (8)	0.0312 (7)	0.0499 (10)	−0.0138 (6)	−0.0176 (7)	0.0173 (7)

### Geometric parameters (Å, º)

**Table:**

Cl1—C4	1.7418 (14)	C6—C7	1.3800 (19)
S1—O2	1.4866 (11)	C6—H6	0.9500
S1—C1	1.7652 (13)	C8—C9	1.4568 (18)
S1—C15	1.7937 (15)	C9—C10	1.3943 (18)
F1—C13	1.3584 (16)	C9—C14	1.4049 (18)
O1—C7	1.3724 (16)	C10—C11	1.385 (2)
O1—C8	1.3745 (15)	C10—H10	0.9500
C1—C8	1.3625 (18)	C11—C12	1.388 (2)
C1—C2	1.4439 (17)	C11—H11	0.9500
C2—C7	1.3945 (18)	C12—C13	1.377 (2)
C2—C3	1.3972 (18)	C12—H12	0.9500
C3—C4	1.3794 (19)	C13—C14	1.372 (2)
C3—H3	0.9500	C14—H14	0.9500
C4—C5	1.402 (2)	C15—H15A	0.9800
C5—C6	1.383 (2)	C15—H15B	0.9800
C5—H5	0.9500	C15—H15C	0.9800
			
O2—S1—C1	107.08 (6)	C1—C8—C9	133.92 (12)
O2—S1—C15	105.95 (7)	O1—C8—C9	115.20 (11)
C1—S1—C15	97.48 (7)	C10—C9—C14	119.61 (12)
C7—O1—C8	106.62 (10)	C10—C9—C8	121.37 (12)
C8—C1—C2	106.92 (11)	C14—C9—C8	119.01 (12)
C8—C1—S1	126.40 (10)	C11—C10—C9	120.64 (13)
C2—C1—S1	126.42 (10)	C11—C10—H10	119.7
C7—C2—C3	119.41 (12)	C9—C10—H10	119.7
C7—C2—C1	105.03 (11)	C10—C11—C12	120.19 (13)
C3—C2—C1	135.54 (12)	C10—C11—H11	119.9
C4—C3—C2	116.67 (12)	C12—C11—H11	119.9
C4—C3—H3	121.7	C13—C12—C11	118.02 (13)
C2—C3—H3	121.7	C13—C12—H12	121.0
C3—C4—C5	123.22 (13)	C11—C12—H12	121.0
C3—C4—Cl1	118.56 (11)	F1—C13—C14	117.86 (13)
C5—C4—Cl1	118.22 (11)	F1—C13—C12	118.28 (13)
C6—C5—C4	120.30 (13)	C14—C13—C12	123.86 (13)
C6—C5—H5	119.8	C13—C14—C9	117.67 (12)
C4—C5—H5	119.8	C13—C14—H14	121.2
C7—C6—C5	116.25 (12)	C9—C14—H14	121.2
C7—C6—H6	121.9	S1—C15—H15A	109.5
C5—C6—H6	121.9	S1—C15—H15B	109.5
O1—C7—C6	125.27 (12)	H15A—C15—H15B	109.5
O1—C7—C2	110.56 (11)	S1—C15—H15C	109.5
C6—C7—C2	124.14 (13)	H15A—C15—H15C	109.5
C1—C8—O1	110.86 (11)	H15B—C15—H15C	109.5
			
O2—S1—C1—C8	−138.71 (12)	C1—C2—C7—C6	−178.69 (13)
C15—S1—C1—C8	112.01 (13)	C2—C1—C8—O1	0.06 (15)
O2—S1—C1—C2	34.63 (13)	S1—C1—C8—O1	174.46 (9)
C15—S1—C1—C2	−74.66 (13)	C2—C1—C8—C9	178.33 (14)
C8—C1—C2—C7	0.43 (14)	S1—C1—C8—C9	−7.3 (2)
S1—C1—C2—C7	−173.96 (10)	C7—O1—C8—C1	−0.54 (14)
C8—C1—C2—C3	−178.15 (15)	C7—O1—C8—C9	−179.16 (11)
S1—C1—C2—C3	7.5 (2)	C1—C8—C9—C10	−30.9 (2)
C7—C2—C3—C4	−0.39 (19)	O1—C8—C9—C10	147.27 (12)
C1—C2—C3—C4	178.04 (14)	C1—C8—C9—C14	150.28 (15)
C2—C3—C4—C5	0.4 (2)	O1—C8—C9—C14	−31.51 (17)
C2—C3—C4—Cl1	−179.30 (10)	C14—C9—C10—C11	−1.3 (2)
C3—C4—C5—C6	−0.2 (2)	C8—C9—C10—C11	179.97 (13)
Cl1—C4—C5—C6	179.54 (11)	C9—C10—C11—C12	0.9 (2)
C4—C5—C6—C7	−0.1 (2)	C10—C11—C12—C13	0.1 (2)
C8—O1—C7—C6	178.70 (13)	C11—C12—C13—F1	178.95 (13)
C8—O1—C7—C2	0.83 (14)	C11—C12—C13—C14	−0.6 (2)
C5—C6—C7—O1	−177.52 (12)	F1—C13—C14—C9	−179.35 (12)
C5—C6—C7—C2	0.1 (2)	C12—C13—C14—C9	0.2 (2)
C3—C2—C7—O1	178.08 (11)	C10—C9—C14—C13	0.7 (2)
C1—C2—C7—O1	−0.78 (15)	C8—C9—C14—C13	179.52 (12)
C3—C2—C7—C6	0.2 (2)		

### Hydrogen-bond geometry (Å, º)

**Table:**

*D*—H···*A*	*D*—H	H···*A*	*D*···*A*	*D*—H···*A*
C11—H11···O2^i^	0.95	2.57	3.3470 (18)	139
C14—H14···O2^ii^	0.95	2.59	3.4884 (17)	157

Symmetry codes: (i) −*x*, −*y*+1, −*z*+1; (ii) −*x*+1, −*y*, −*z*+1.
