# Supplementary figures and images for: Crystal structure of 5-chloro-2-(3-fluoro­phen­yl)-3-methyl­sulfinyl-1-benzo­furan
Source: Acta Crystallogr Sect E Struct Rep Online. 2014 Aug 9;70(Pt 9):o991–2. doi: 10.1107/S1600536814017966 (PMC4186186; doi:10.1107/S1600536814017966)

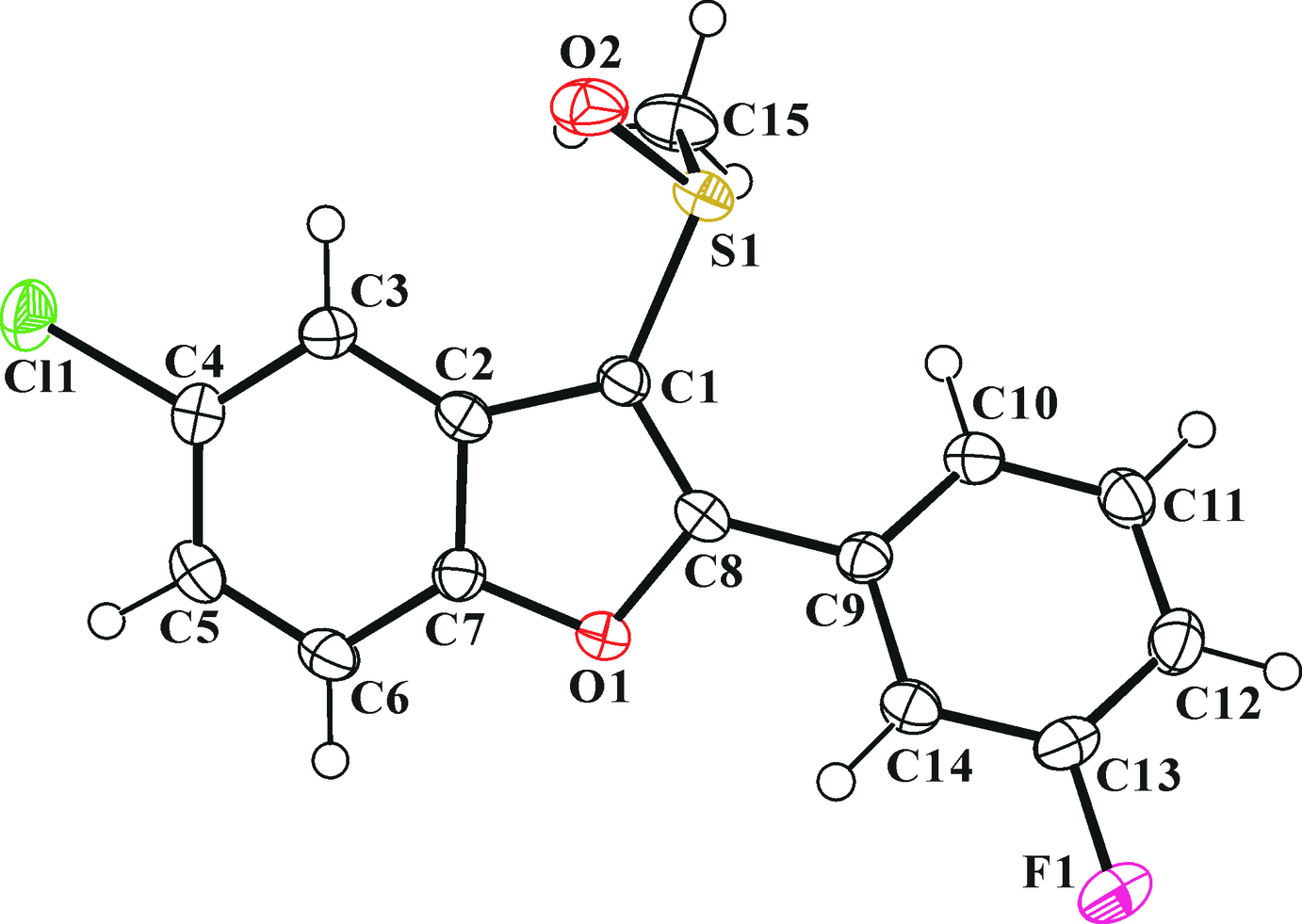

Supplement: Supplementary file 4 [file e-70-0o991-fig1.tif]

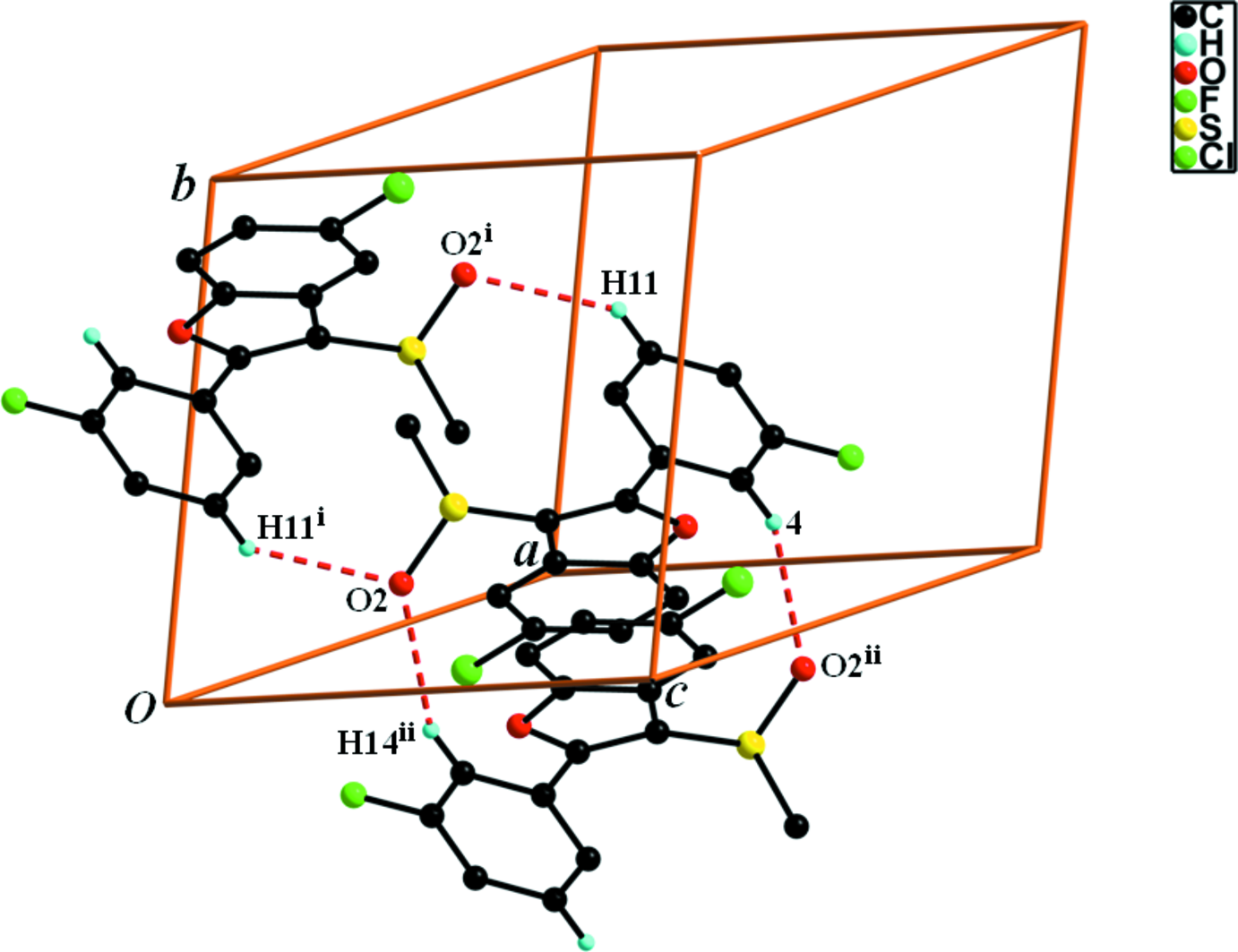

Supplement: Supplementary file 5 [file e-70-0o991-fig2.tif]
